# Improving Diet Quality of People Living With Obesity by Building Effective Dietetic Service Delivery Using Technology in a Primary Health Care Setting: Protocol for a Randomized Controlled Trial

**DOI:** 10.2196/64735

**Published:** 2025-04-30

**Authors:** Deborah A Kerr, Clare E Collins, Andrea Begley, Barbara Mullan, Satvinder S Dhaliwal, Claire E Pulker, Fengqing Zhu, Marie Fialkowski, Richard L Prince, Richard Norman, Anthony P James, Paul Aveyard, Helen Mitchell, Jacquie Garton-Smith, Megan E Rollo, Chloe Maxwell-Smith, Amira Hassan, Hayley Breare, Lucy M Butcher, Christina M Pollard

**Affiliations:** 1 Curtin School of Population Health Curtin University Bentley Australia; 2 Curtin Medical Research Institute Curtin University Bentley Australia; 3 School of Health Sciences College of Health, Medicine and Wellbeing University of Newcastle Callaghan Australia; 4 Food and Nutrition Research Program Hunter Medical Research Institute New Lambton Heights Australia; 5 Curtin Enable Institute Curtin University Bentley Australia; 6 Office of the Provost Singapore University of Social Sciences Singapore Singapore; 7 Health & Social Sciences Singapore Institute of Technology Singapore Singapore; 8 Obstetrics & Gynaecology Academic Clinical Program Duke NUS Medical School National University of Singapore Singapore Singapore; 9 Nutrition and Health Innovation Research Institute School of Medical and Health Sciences Edith Cowan University Joondalup Australia; 10 School of Electrical and Computer Engineering Purdue University West Lafayette, IN United States; 11 Population Sciences in the Pacific Program University of Hawaii Cancer Center Honolulu, HI United States; 12 The University of Western Australia Medical School The University of Western Australia Nedlands Australia; 13 Nuffield Department of Primary Care Health Sciences University of Oxford Oxford United Kingdom; 14 Hope Community Services Perth Australia; 15 Health Networks Clinical Excellence Division Department of Health WA East Perth Australia; 16 Community and Population Health East Metropolitan Health Service Perth Australia

**Keywords:** obesity, diet, diet quality, digital behavioral interventions, health behavior change, mHealth, mobile health, eHealth, mobile food record, telemedicine, weight management, obese, RCT, randomized controlled trial, Australia, adult, nutrition, dietitians, access, dietary management, intervention, digital health

## Abstract

**Background:**

Almost a third of Australian adults are living with obesity, yet most cannot access medical nutrition therapy from dietitians, that is, the health professionals trained in dietary weight management services. Across the health system, primary care doctors readily identify people who may benefit from weight management services, but there are limited referral options in the community. Dietitians are trained to provide evidence-informed dietary treatment of overweight and obesity but are underutilized and underresourced. The chat2 (Connecting Health and Technology 2) trial will test combining new technologies for dietary assessment with behavior change techniques to improve outcomes for people living with obesity.

**Objective:**

This study aimed to compare the effectiveness of a 1-year digital dietary intervention, with standard care on body weight reduction and improved diet quality, in adults living with obesity delivered by dietitians in a primary care setting.

**Methods:**

This randomized controlled trial will compare a 1-year, digitally tailored, feedback dietary intervention with a control group in 430 adults living with obesity (BMI≥30 to ≤45 kg/m^2^). Participants will be recruited by letters sent to individuals randomly selected from the electoral roll and supplemented by hospital site posters, newsletters, and unaddressed mailbox delivery postcards sent to residential street points. The primary outcome is change in body weight, measured face-to-face at a baseline, 6 months, and 12 months. A 4-day, image-based dietary assessment tool (mobile Food Record) will be used to measure diet quality score. Secondary outcomes include diet quality score; dual-energy absorptiometry body composition; and total cholesterol, triglyceride, low-density lipoprotein, high-density lipoprotein, glycated hemoglobin, and fasting glucose levels. The intervention group will receive 8 video counseling sessions with a trained dietitian delivered over 12 months to support dietary behavior change and relapse prevention. The trial is unblinded. Both groups will receive feedback on their clinical chemistry and dual-energy absorptiometry scans at each time point.

**Results:**

Participant recruitment commenced in July 2023 and ended in August 2024. Data analysis will commence in 2025, with the anticipated publication of results in 2026.

**Conclusions:**

If found to be effective, the results of this randomized controlled trial will support the delivery of effective, evidence-based weight management advice using new technologies. Improving community access to high-quality dietetic services will ensure more effective use of the dietetic workforce to improve outcomes for people living with obesity.

**Trial Registration:**

Australian New Zealand Clinical Trials Registry ACTRN12622000803796; https://anzctr.org.au/Trial/Registration/TrialReview.aspx?id=383838

**International Registered Report Identifier (IRRID):**

DERR1-10.2196/64735

## Introduction

The high and increasing prevalence of obesity across the world is a major challenge for health care delivery and confers a substantial burden on individuals and the health care sector. The effects of obesity are widely recognized as one of the leading health concerns, affecting all age and socioeconomic groups [1]. For example, in Australia, overweight and obesity and dietary risks collectively account for nearly half of the 38% of the preventable burden of disease, with overweight and obesity being the leading contributor to nonfatal burden [2]. In tandem, direct health care costs associated with obesity-related conditions, such as cardiovascular disease, respiratory disease, type 2 diabetes, and some cancers, are rising with an unequal distribution corresponding to the level of socioeconomic disadvantage [3,4]. Almost a third (31%) of Australian adults are living with obesity, yet most are unable to access weight management services. An urgent priority for health service delivery is to identify the most cost-effective approach to delivering evidence-based weight management.

Primary care is a critical component of addressing obesity and requires a person-centered approach to treatment [5]. This includes changing the weight-stigmatizing narrative among health professionals working with people living with obesity [6]. Treatment guidelines for obesity are evaluated by the person’s BMI and the severity of obesity-related complications [5]. Intensive interventions involving very low-energy diets, pharmacology, or bariatric surgery are indicated with BMI≥40 kg/m^2^ and obesity-related complications [5]. Whereas, for people with BMIs between 30 and 40 kg/m^2^ without complications, supervised lifestyle interventions to achieve 10% initial weight loss are recommended [5]. However, unfortunately in Australia, there are limited referral options for weight management in the community. Publicly supported bariatric surgery waiting lists are long and triaged to those with a BMI≥40 kg/m^2^ with obesity-related complications, and while recently pharmacological treatments have been increasingly used in the treatment of obesity [7], issues with availability and cost limit the access to these pharmacological treatments. In addition, not everyone has access to or wishes to choose the pharmacological pathway. Therefore, continued research on behavioral lifestyle interventions is needed as they remain the first-line treatment for people living with obesity [7].

Lifestyle modification is a fundamental component of weight management. The most effective interventions to date have used behavioral strategies to support people to achieve energy reduction [8]. Guidelines for comprehensive weight management interventions identify the importance of personal counseling to achieve a clinically significant 5% weight loss to confer health benefits, including lowering blood pressure or delaying progression to type 2 diabetes [9]. In a review of primary care interventions for obesity, intensive counseling (≥12 sessions per year), delivered face-to-face, by phone, or electronically, produced weight loss of 4-7 kg, compared with low-dose counseling (≤12 sessions per year) without behavioral strategies, which only resulted in only modest losses of 1-2 kg [8], supporting the value of higher intensity behavioral interventions.

Behavioral interventions for weight management are complex and may include a range of components and modes of delivery. Factors associated with greater effectiveness include changes in diet, provision of meal replacements, and whether the program was delivered by a dietitian or psychologist [10]. A systematic review of weight management interventions demonstrated that those provided by a dietitian resulted in improvements in BMI and other cardiometabolic outcomes compared with control conditions [11]. Behavioral strategies of these weight management interventions, however, were not explored. Teasing out the behavioral components of interventions is important to discover the most effective behavior change techniques. In a review of behavioral interventions in type 2 diabetes, frequent feedback and support were associated with weight reduction [12].

To date, few studies have examined the combined effectiveness of behavior change techniques with new cutting-edge digital technologies and medical nutrition therapy. Digital technologies are effective in supporting weight loss, particularly when combined with personalized feedback [13]. One of the challenges for dietitians in working with individuals living with obesity is the ability to easily and accurately assess diet and dietary behaviors—an important component of personalized advice. Another consideration is people’s willingness to record their dietary intake. An image-based dietary record app with time and date stamp data, termed the mobile food record (mFR), has been shown to be feasible for dietary assessment and feedback, a key component for changing dietary behaviors [14,15]. The content of the images is confirmed either by the participant or by novel automated methods using computer vision and machine learning techniques under development [16,17]. An additional technological intervention feature is the use of videoconferencing. Improving access to weight management advice, through videoconferencing, may assist people in managing their weight, but is yet to be fully evaluated. This study will provide the first test of these digital technologies in people living with obesity in Australia. The study is designed based on key components of effective weight management interventions that include behavioral strategies guided by the (1) capability, opportunity, motivation, and behavior (COM-B) model [18]; (2) the behavior change techniques; and (3) self-monitoring, goal setting, and individually tailored feedback on dietary behaviors [15,19-21]. The objective is to compare the effectiveness of a 1-year digital dietary intervention on body weight reduction and improved diet quality, in adults living with obesity delivered by dietitians in a primary care setting, with a control group.

## Methods

### Study Design

**Figure 1:**
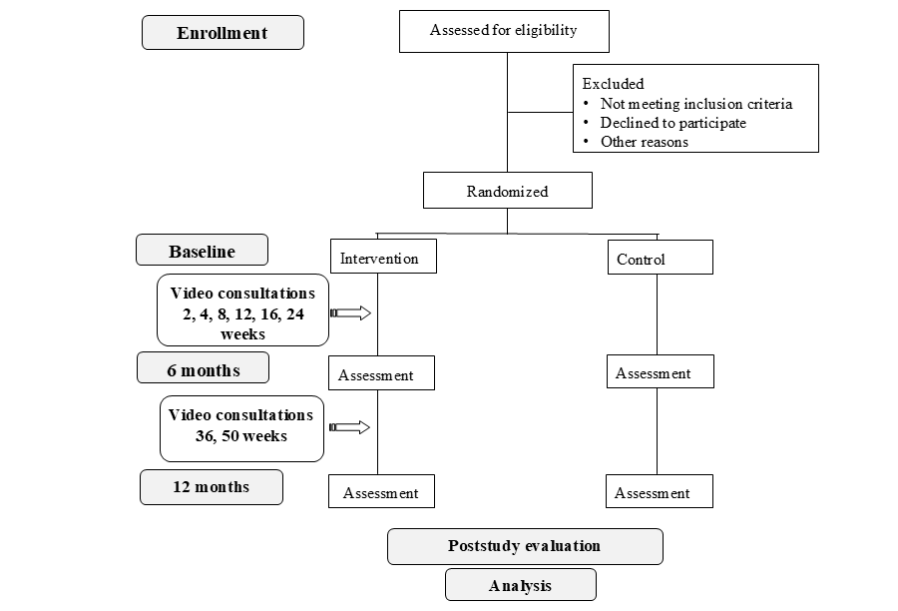
Study design with randomization to two groups: intervention and control groups.

The chat2 (Connecting Health and Technology 2) study is a 1-year randomized controlled trial (RCT). Consenting participants will be randomly assigned (1:1) to an intervention or minimal intervention control group. Figure 1 shows the CONSORT (Consolidated Standards of Reporting Trials) study design. Assessments will occur at 0, 6, and 12 months with the primary outcome assessed at 12 months.

### Consumer Engagement

Engaging with health consumers alongside doctors and health professionals in weight management is a novel and strategic approach to this complex health issue. Using a systems approach provides opportunities for people with lived experience, health professionals, and policy makers to work together to take positive action to address the complex issue of weight management. A systems science group model–building approach was used to develop the East Metropolitan Health Service (EMHS) Obesity Prevention Strategy, to cocreate a shared understanding of the complex issue of obesity, its causes and consequences, and feasible solutions [22]. Health service executives; clinicians; allied health professionals; and representatives from community service providers, government, and nongovernment agencies identified the gap in services for people living with obesity and prioritized action to address this.

This intervention design was developed in partnership with EMHS and informed by the Western Australian Healthy Weight Action Plan, led by the Western Australian Health Department’s partnership with the Western Australian Primary Health Alliance and the Western Australian Health Consumers’ Council [23]. This partnership ensured that peoples’ lived experiences were central to the research. Since 2018, the Western Australian Health Consumers’ Council has conducted several engagement activities to ensure the voices of people with lived experience of overweight and obesity were key drivers of change. A review of weight-management services by this group identified gaps in service delivery, including the lack of supported behavior change programs for weight management, the absence of clear referral pathways, and limited access to a skilled workforce in weight-management care. This study will recruit a panel of health consumers who have lived experiences of overweight and obesity as members of the project advisory board to ensure that their perspectives and voices are considered at all stages of the project. Their role will include reviewing all study materials, including ethics documents, study website design, and recruitment strategies and materials. In addition, the project team and consumers will work to ensure that the trial is respectful, recognizing that participants are likely to have experienced the negative consequences of weight stigma on their health [6].

### Setting

The study setting will target recruitment in the EMHS catchment, 1 of 4 publicly funded health service areas in Perth, Western Australia. EMHS is responsible for the maintenance and improvement of the well-being and health of 725,000 people residing within its catchment, approximately one-third of the population of Western Australia. This is achieved through the provision of an extensive health network including tertiary, secondary, specialist, community, and population health services. The EMHS catchment has the highest prevalence of overweight and obesity in the Perth metropolitan area, where 77% of adults live with overweight or obesity (43% live with obesity) [24,25].

### Recruitment

#### Overview

Participants will be recruited by letters sent to individuals randomly selected from the electoral roll in Perth to provide representation across all socioeconomic status groups. Electoral roll recruitment is a feasible and effective recruitment method previously used by the research team [15]. The mailout will be staggered so that recruitment occurs over 12 to 18 months with a target of 25 participants per month. This will ensure adequate resourcing and staffing for the study. Other recruitment methods will supplement the mail out and will include EMHS hospital sites displaying posters and recruitment postcards; newsletters sent to primary care doctors and practice managers; EMHS and university newsletters; and unaddressed mailbox delivery to residential street points in suburbs located in the EMHS catchment area. Quota sampling will ensure equal numbers between study groups and genders. After receiving the letter of invitation, those who wish to take part in the study will contact the research team by email, mobile telephone (text or voice), or the study website, where they will be directed to read the information statement and consent online, and complete an online screening questionnaire. Computer literacy was not an explicit criterion to be eligible for the study.

To be eligible, participants must be aged 18-65 years, have a self-reported BMI≥30 to ≤45 kg/m^2^, own a mobile telephone with internet access, and be able to attend the study center in metropolitan Perth for all 4 visits. Participants will be excluded on the basis of serious illness or medical conditions including diabetes requiring insulin; medical dietary restrictions (eg, low potassium diet for renal failure); receiving counseling from a dietitian; had or planned to have a surgical intervention for weight management in the next year; severe heart conditions (eg, cardiac failure requiring fluid restriction and a stroke or myocardial infarction in the previous 6 months); severe life-limiting illness receiving palliative treatment (eg, cancer, cirrhosis, and pulmonary disease or heart failure); currently taking or plan to take weight loss medication in the next 12 months; pregnancy or breastfeeding, or planning to become pregnant or breastfeed in the next year; receiving treatment for an eating disorder; or unable to participate in telehealth dietary consultations.

#### Randomization

Block randomization will be used with allocation concealment from the active research team via the use of sealed opaque envelopes. The randomization will be in blocks of 4, with stratification by gender (men, women, and other), where participants will be assigned to either the intervention or minimal intervention control groups (1:1). Sequence generation will be conducted before the commencement of the study by a statistician using a randomization table created in Stata (version 18, StataCorp). The electronic file will be kept in a secure password-protected server by the statistician. Randomization will occur at the second study visit to assign participants to either the intervention or control groups. On opening the randomization envelope, the participant will inform the research staff which group they are allocated to. The trial is unblinded due to the nature of the intervention; it is not possible to blind participants or researchers to the intervention group after allocation.

#### Data Collection Procedures

Eligible participants will be notified via email and invited to attend 2 baseline in-person visits 1 week apart at the Curtin University study center. Before the first visit, participants will be asked to complete an online questionnaire (including ethnicity, education, employment, postcode, alcohol use, smoking, physical activity, eating and dietary habits, motivation, and weight loss history), as detailed in Table 1. At the baseline visit, participants will be asked to fast overnight before a blood test, whole-body composition dual-energy absorptiometry (DXA) scan, and height and weight measurement. They will receive training on how to record their dietary intake by taking images using the mFR app, a mobile app developed by Purdue University. The mFR app will be installed on participants’ mobile devices (Android [Google] or iPhone [Apple Inc]) at no cost. They will be asked to return approximately 1 week later, where they will receive a copy of their DXA scan and blood tests. They will be interviewed by the research dietitian to clarify the content of their mFR images and probe for missing data. Participants will undergo random allocation to the intervention or control groups. The assessments will be repeated for both groups at 6 and 12 months (Table 1). Participants will be asked to keep an adverse events diary (eg, hospital admissions, illness, or changes to medications) for the duration of the study that will be reviewed at the 6- and 12-month study assessment visits. Participants will be encouraged to discuss their involvement with their primary care doctor, who will be notified by the research team that their patient has volunteered and has been provided with blood test results, information about the trial, and contact details of the research team via a secure facsimile. Before each appointment, participants will be sent an email with details of their visit, along with a text message reminder the day before. Both intervention and control group participants will be provided with a study booklet with information on healthy eating from the Australian Dietary Guidelines (ADG) website [26]. This will include healthy eating information on food groups, serving sizes, links to recipes, and label reading.

**Table 1:** Frequency of assessment variables for the chat2 (Connecting Health and Technology 2) study intervention and control groups.

Variables	Group	Baseline	6 months	12 months
Height and weight (measured at clinic visit)	I^a^ and C^b^	Yes	Yes	Yes
Body composition: whole body dual-X-ray-absorptiometry scan of regional or total body fat (including visceral body fat)	I and C	Yes	Yes	Yes
Adverse events diary	I and C	Yes	Yes	Yes
Dietary intake: 4-day mFR^c^	I and C	Yes	Yes	Yes
mFR usability to assess user feedback and method preference [14,27]	I and C	Yes	Yes	Yes
Blood sample: total cholesterol, triglyceride, LDL^d^ and HDL^e^ cholesterol, fasting glucose, and glycated hemoglobin	I and C	Yes	Yes	Yes
Health status EQ-5D, a 5-item scale to assess utility and health-related quality of life [28]	I and C	Yes	Yes	Yes
Sociodemographic and personal characteristics assessed via questions on sex, age, eating behavior, educational level, country of birth, ethnicity, socioeconomic status, and financial status	I and C	Yes	Yes	Yes
18-item Food Insecurity [29]	I and C	Yes	No^f^	No^f^
Self-report Habit Index Score [30]	I and C	Yes	Yes	Yes
Creature of Habit questionnaire [31]	I and C	Yes	Yes	Yes
Self-reported physical activity assessed via the International Physical Activity Questionnaire (short form) [32]	I and C	Yes	Yes	Yes
Depression, Anxiety, Stress Scale, with 21 self-report items to assess severity of depression, anxiety, and stress [33]	I and C	Yes	Yes	Yes
Weight-Loss History, an 8-item tool to assess previous weight-loss history [34]	I and C	Yes	No^f^	No^f^
The Health Care Climate Questionnaire [35]	I	No^f^	No^f^	Yes
Personalized Nutrition Questionnaire [21]	I	Yes	Yes	Yes

^a^I: intervention.

^b^C: control.

^c^mFR: mobile Food Record.

^d^LDL: low-density lipoprotein.

^e^HDL: high-density lipoprotein.

^f^Data not collected.

#### Dietary Assessment and Analysis

**Figure 2:**
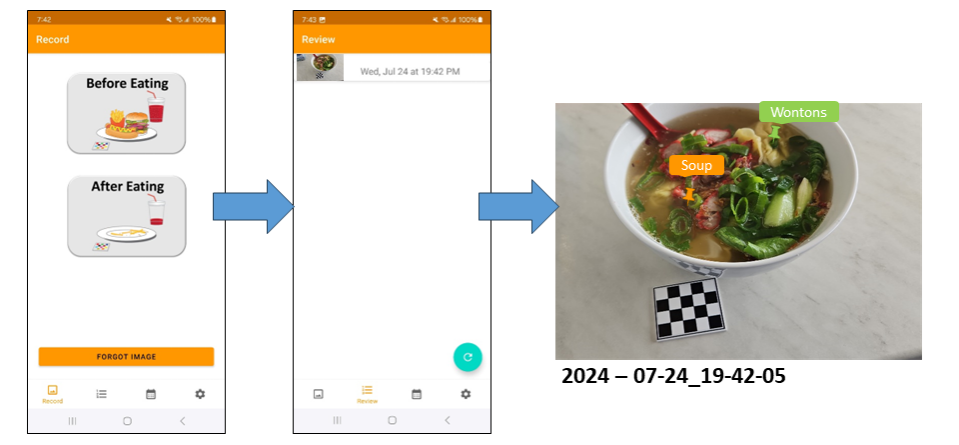
Review process for viewing a labeled eating occasion, with colored labels identifying the foods and the time and date stamp displayed.

Participants will be provided with access to the mFR app on their mobile device to record their dietary intake at baseline, 6 months, and 12 months (Figure 2) [16,36]. They will be asked to take “before eating” and “after eating” images of all foods and beverages consumed over 4 consecutive days, including 1 weekend day. The mFR app communicates with a dedicated cloud-based server for the storage and processing of images via Wi-Fi or 4G and 5G networks. A fiducial marker is included in the image to allow improved accuracy for quantifying dietary intake [37-39]. The mFR app will be updated regularly in response to operating systems updates (Android and iPhone). Participants will label the content of their images, referred to as mini labels. When the participant starts typing the word, a list of foods will appear where they can select from a list of approximately 800 food and beverage items. These labels link to a food composition database (not visible to the participant) with the food code, detailed description, and energy and nutrient composition [40]. These mini labels will be linked to a code for estimating food group servings to enable a food-based assessment. At the study visit, the research dietitian will review the images with participants through the web and probe for food and beverage items not recorded (Figure 2). The data from the mFR will be entered into a purpose-built Microsoft Access database platform to assess the food group servings.

#### Diet Quality Scoring

Analysis of the 4-day mFR will be undertaken by the research dietitian, and a food-based diet quality score (DQS) will be calculated (Table 2). In addition to energy reduction, this study will assess diet quality, as there is emerging evidence that diet quality, particularly reduced ultraprocessed foods, have a role in energy balance [41,42]. The 11-component DQS is based on the ADG [26] and adapted from 2 food-based indexes developed for Australian adults [43,44]. The DQS uses a continuous weighting system based on increments of food group servings, or proportions of total intake for behaviors without set recommendations, such as variety of fruits and vegetables. For core food groups, such as fruits and vegetables, the maximum weighting assigned to the item is determined by the ADG-recommended daily servings. To achieve weight reduction consistent with study goals, discretionary foods and beverages will be split into separate components. Reverse scoring is assigned for discretionary (energy-dense and nutrient-poor) foods, sugar-sweetened beverages, and alcoholic beverages, where a maximum score of 10 points is achieved with zero servings. The DQS at baseline and 6 months will be provided to the study dietitians to be relayed to intervention group participants in the video consultations.

**Table 2:** Diet quality scoring calculated from average daily servings based on the Dietary Guidelines for Australia. Total maximum score of 100 points from 11 components.

Item Description	Minimum score	Composite scores	Maximum score
Fruit	0 to <0.5 servings (0 points)	≥0.5 to <1.0 serving (2.5 points) ≥1.0 to <1.5 servings (5 points) ≥1.5 to <2.0 servings (7.5 points)	≥2 servings (10 points)
Vegetables and legumes or beans	0 to <1.0 serving (0 points)	≥1.0 to <2.0 (2 point) ≥2.0 to <3.0 servings (4 points) ≥3.0 to <4.0 servings (6 points) ≥4.0 to <5.0 servings (8 points)	≥5 servings (10 points)
Variety of fruits	0 type (0 points)	1 type (1 point) 2 types (2 points) 3 types (3 points) 4 types (4 points)	5 or more types (5 points)
Variety of vegetables	0 type (0 points)	1 type (1 point) 2 types (2 points) 3 types (3 points) 4 types (4 points)	5 or more types (5 points)
Lean meat, poultry, fish, eggs, nuts, seeds, legumes, or beans (women)	>2.5 servings or <0.5 servings (0 points)	≥0.5 to <1.0 (2 points) ≥1.0 to <1.5 (4 points) ≥1.5 to <2.0 (6 points) ≥2.0 to <2.5 (8 point)	2.5 servings (10 points)
Lean meat, poultry, fish, eggs, nuts, seeds, legumes, or beans (men)	>3 servings or <1.0 serving (0 points)	≥1.0 to <1.5 servings (2 points) ≥1.5 to <2.0 servings (4 points) ≥2.0 to <2.5 servings (6 points) ≥2.5 to <3.0 servings (8 point)	3 servings (10 points)
Milk, yoghurt, cheese, and alternatives	<0.5 servings (0 points)	≥0.5 <1.0 servings (2 points) ≥1 to <1.5 servings (4 points) ≥1.5 to <2.0 servings (6 points) ≥2 to <2.5 servings (8 points)	≥ 2.5 servings (10 points)
Grain (cereal) foods, mostly wholegrain, and high cereal fiber varieties	<1.0 serving or >6.0 servings (0 points)	≥1.0 to <2.0 servings (1 point) ≥2.0 to <3 servings (2 points) ≥3.0 to <4 servings (5 points) ≥4.0 to <5 servings (6 points) ≥5.0 to <6 servings (8 points)	6.0 servings (10 points)
Discretionary foods	≥2.75 servings (0 points)	≥1.75 to <2.75 servings (2 points) ≥0.75 to <1.75 servings (4 points) >0 to <0.75 servings (8 points)	0 servings (10 points)
Sugar-sweetened beverages	≥2 servings (0 points)	≥1.25 to <2 servings (2 points) ≥0.5 to <1.25 servings (4 points) >0 to <0.50 servings (8 points)	0 servings (10 points)
Alcoholic beverages	≥2 servings (0 points)	≥1.25 to <2 servings (2 points) ≥0.5 to <1.25 servings (4 points) >0 to <0.50 servings (8 points)	0 servings (10 points)
Plain water	<1 cup (0 points)	≥1 to 3 cups (2 points) ≥3 to 5 cups (6 points) ≥5 to <8 cups (8 points)	≥8 cups (10 points; women) ≥10 cups (10 points; men)

#### Intervention Features

The intervention features, content, and behavior change techniques to be implemented are shown in Table 3. The selection of intervention features and strategies is guided by the COM-B model [18]. The COM-B model identifies behavioral targets when developing interventions and focuses on 3 factors required to change behavior: capability, opportunity, and motivation. Performing a behavior requires individuals to be capable or have physical and psychological abilities (eg, nutrition knowledge and cooking skills). Opportunity includes both practical and social aspects (eg, access to healthy foods, supportive environments, and social norms). Motivation comprises automatic drivers like habits as well as beliefs, emotions, impulses, and reflective processes such as intentions and planning. Behavior change techniques will include feedback, self-monitoring, feedback on performance, goals or planning, repetition, and substitution or habit formation [20].

**Table 3:** Overview of the study goals and intervention features, content, and behavior change techniques.

Goal	How to achieve	Behavior change techniques [20]
1. Achieve a reduction in body weight by at least 5% in 12 months	Avoid or limit discretionary foods, sugary drinks, and alcohol Eating less at meals and snacks (except for vegetables and salad) Eating less often (avoid snacking—except for vegetables and salads)	Feedback—DQS^a^ from mFR^b^
2. Improve DQS	Eat from each of these five food groups with more variety (especially vegetables and fruit) every day Vegetables and legumes/beans, including different types and colors Fruit—choose fresh fruit as much as possible Grains (cereal) foods, mostly wholegrain and high cereal fiber varieties Lean meats and poultry, fish, eggs, tofu, nuts, and seeds, and legumes/beans (eg, kidney beans, chickpeas, lentils, and other beans) Milk, cheese, yoghurt, and alternatives, mostly reduced fat Drink more plain water (no added flavors); 2.1-2.6 L/day (8-10 cups)	Feedback and self-monitoring—DQS from mFR
3. Behavior change	Find your motivation Plan to make changes Set SMART^c^ goals Build healthy habits	Questionnaire for current barriers and enablers to eating behavior change and some goals (PNQ^d^) Goals and planning—action planning, commitment, goal setting, problem solving Goals and planning—action planning, commitment, goal setting problem solving, coping planning, implementation intentions Repetition and substitution—habit formation

^a^DQS: diet quality score.

^b^mFR: mobile food record.

^c^SMART: Specific, Measurable, Achievable, Relevant, and Time-Bound.

^b^PNQ: Personalized Nutrition Questionnaire.

#### Intervention Group

Participants allocated to the intervention group will be offered 8 video consultations (approximately 20 to 45 minutes based on intervention stage) with a dietitian for the first 6 months (at 2, 4, 8, 12, 16, and 24 weeks) to provide support and guidance for unhelpful behaviors (eg, emotional eating) and additional maintenance calls (relapse prevention and coping planning) at 9 and 12 months (8 in total). This number of individual contacts was chosen as a balance between recommendations [9] and practical service delivery costs. Before selecting telehealth consultations, participants complete the Personalized Nutrition Questionnaire (PNQ) to identify and prioritize self-perceived factors impacting on their eating behaviors [21]. The PNQ is a tool designed to support the dietitian during consultations by collecting information on barriers before the session. Based on the COM-B framework [18], the PNQ has been used with a variety of groups to support dietitian-led telehealth consultations [45-47]. The medical nutrition therapy consultation will cover dietary feedback focusing on their DQS, goal setting, and behavioral strategies. Feedback will be formulated from the analysis of the mFR and will focus on key dietary messages (Table 3). The content will address each participant’s personal barriers to changing dietary behaviors, reinforce capability, motivation, and guide adoption of health-enhancing habits.

#### Control Group

The control group will complete all measures and will be provided with a standard care study booklet using information from the ADG website [26]. At the end of the study, control group participants will receive personalized feedback on their outcomes, including their dietary feedback report.

### Dietetics Training and Consultations

Research dietitians will undertake 8 hours of in-person training, before their consultations with participants. The training will include approximately 2 hours of self-paced, recorded, online training in the dietitian’s own time and 6 hours of online or in-person workshops. The training will include current evidence-based practice for people living with obesity, addressing weight bias or stigma, study design, ethics, mFR technology or study systems, documentation, and fidelity of implementation adherence. Study dietitians will complete online training in motivational interviewing in health care [48]. The behavior change training conducted by the health psychology team will be guided by the COM-B model, and embedded with behavior change techniques [49]. A follow-up booster session will be conducted midway through the intervention. Standard guides will be developed for each of the 8 consultation sessions by the research team. Dietitians will use the consultation guide for each session, record attendance, and contact participants for missed sessions. The style of communication will be consistent with self-determination theory and motivational interviewing principles [15,50,51]. Fidelity of implementation adherence will be guided by a conceptual framework for implementation fidelity [52] (ie, adherence to the guide as prescribed and quality of delivery) and will be assessed for dietitians using measures of fidelity and engagement [53]. Practice management software for allied health professionals [54] will be used to manage appointments; send text appointment reminders; document attendance; record treatment notes; and access anthropometry, DQS, PNQ, and blood test results. Study dietitians will document each of the 8 sessions according to the nutrition care process: nutrition assessment, nutrition diagnosis, nutrition intervention, nutrition monitoring, and evaluation [55,56]. They will also document behavior change techniques implemented according to the COM-B model [20].

### Process Evaluation

#### Overview

A process evaluation will be undertaken to evaluate the implementation and impact of the intervention (ie, fidelity, dose, and reach) using the UK Medical Research Council evaluation framework [57]. Adherence to the intervention will be assessed by attendance at sessions as documented in the practice management software and set at 75% attendance. Delivery of behavior change elements will be assessed by reviewing the dietitian’s documentation of behavior change techniques. Additional components relevant for digital interventions will assess acceptability, engagement, effectiveness, and sustainability. A questionnaire will be used to evaluate participants’ perception of the intervention (ie, dietetic video consults, feedback, usefulness of advice, suitability, and relevance to age group). Both intervention and control participants will evaluate their perceptions of intervention features (ie, receiving results of blood tests and whole-body DXA scans).

#### Poststudy Qualitative Interviews

At the completion of the study, approximately 20 semistructured interviews will be conducted with intervention and control participants using videoconferencing. The interviews aim to assess participant experiences, the intervention impact, and perceptions of various strategies and study materials. The interview topic guide will be developed using the COM-B model as a theoretical framework to explore participants’ capability, opportunity, and motivation to change their eating behaviors [18]. The interviews will be audio recorded and transcribed verbatim. Data will be analyzed using framework analysis, a systematic approach that provides a structured method for managing and interpreting interview data [58,59]. While the COM-B model will be used to inform the development of the topic guide, the analysis will take an inductive approach to ensure that themes generated from participants’ experiences are captured beyond the predefined theoretical framework. The procedure for analysis will involve (1) verbatim transcription, (2) familiarization with the data through reading of transcripts, (3) open coding data segments from transcripts, (4) developing an analytical framework based on generated codes, (5) coding all transcripts using the analytical framework, (6) charting the data into a framework matrix, and (7) interpreting the data through pattern mapping and theme generation. The analysis will be facilitated using NVivo (Lumivero) qualitative data analysis software. Several strategies will be implemented to ensure qualitative rigor. A reflexive journal will be maintained throughout data collection and analysis to document assumptions, potential biases, and evolving interpretations. Regular team debriefing sessions will offer opportunities to discuss and challenge interpretations. Participants will be offered the opportunity to review their interview transcripts and provide feedback on initial interpretations through member checking. Together, these strategies will support the credibility and confirmability of the findings [60]. Dependability will be enhanced through clear documentation of the analytical process in NVivo, with an audit trail capturing analytical decisions and interpretation development [60]. To support transferability, detailed descriptions of the context, participant characteristics, and the RCT will be provided. Findings will be reported using the Consolidated COREQ (Criteria for Reporting Qualitative Research) checklist to ensure comprehensive and transparent reporting of the research process [61].

### Statistical Analysis

#### Overview

The primary outcome is a change in weight at 12 months for both groups. Secondary outcome variables are changes in DQS, body fat (DXA derived), total cholesterol, triglyceride, low-density lipoprotein and high-density lipoprotein cholesterol, fasting glucose, and glycated hemoglobin. It is hypothesized that at 12 months the intervention group will achieve at least a 5% reduction in body weight. Data on change in outcome variables in each of the 2 groups will be compared using general linear models after adjusting for covariates. Assumptions of the analyses will be assessed by examining residuals. Data will be transformed if the assumptions of the analyses are not satisfied. Possible covariates will include age, sex, country of birth, ethnicity, highest education level, socioeconomic index for area, and baseline value of the variable analyzed. *P* values <.05 will be considered statistically significant. Effect size of differences between groups will be expressed as adjusted mean difference and associated 95% CIs. Data on change in outcome variables postintervention and follow-up time points will also be converted into binary categorical variables and analyzed using multivariable logistic regression and generalized estimating equations. The odds ratio and associated 95% CIs will be reported.

#### Sample Size

A sample size of 342 participants (n=171 per group) will have 90% power to detect a difference in change between arms of at least 5% of body weight at 12 months between groups, using a conservative estimate of SD, at 90% power and 5% level of significance. Assuming 20% (n=86) of participants are not followed up, this would necessitate 430 (215 in each group) participants to be recruited. Difficulties in obtaining a sufficient number of participants in weight management trials are well documented [11]. Assuming an 8% change in weight and 20% dropout would require 170 participants. Estimates of means and SDs, including percentage dropout, were obtained from existing trial data for this study’s power calculation.

#### Economic Evaluation

A stepped cost-effectiveness analysis of the 2 arms (intervention with control) will be conducted. The economic evaluation will consider the relative costs and outcomes of the intervention from the health and social care perspective. For the final step, to facilitate a cost-utility analysis, the EQ-5D will be administered to capture the quality of life [28]. The 5-level version more sensitively captures changes in health-related quality of life. Incremental quality-adjusted life years will be estimated for both groups. Incremental costs, collating the time needed to provide tailored advice, medication, and self-estimated weekly food expenditure will allow economic evaluation from the perspective of the health system and the broader society (by considering all costs). Univariate and multivariate sensitivity analyses will be undertaken. In particular, the impact of different methods of extrapolating costs and outcomes beyond the horizon of the trial will be assessed. The costing model will include resources required to assess ongoing maintenance of the mFR, including changes because of upgrades to operating systems. The major cost of the intervention is likely to be the provision of dietetic counseling (research personnel and research platform costs). This will be estimated by recording time spent deconstructing the mFR data and then interpreting data and constructing appropriate feedback.

### Ethical Considerations

The project protocol has been approved by the Curtin University Human Research Ethics Committee (approval HRE2022-0059) and the Department of Health WA Human Research Ethics Committee (PRN RGS0000005490). The trial was registered with the Australian New Zealand Clinical Trials Registry (ACTRN12622000803796).

Informed consent to participate in the study will be received during the initial recruitment stage before any data collection. Volunteers who are interested in participating will follow a Qualtrics survey link to the participant information sheet that details their involvement in the trial. Participants will be provided with a copy of the participant information sheet at their first face-to-face visit. Following this information, participants will be asked to consent to participate in the study and informed that they have the right to withdraw from the study at any time without penalty. Participants will receive an Aus $20 (~US $12.70) grocery store voucher at each face-to-face visit.

## Results

Participant recruitment commenced in July 2023 and ended in August 2024. Recruitment was staggered over 12 to 14 months. Data analysis will commence in 2025, with the anticipated publication of results in 2026.

## Discussion

### Principal Outcomes

This RCT will compare the effectiveness of a 1-year, digitally tailored, dietitian-delivered, feedback dietary intervention with standard care in adults living with obesity (BMI≥30 kg/m^2^) in a primary care setting. Specifically, this project addresses a gap in dietetics service for the delivery of telehealth weight management care in the community. A key outcome of this research is to improve the primary care referral pathway for patients living with obesity by improving community access to high-quality dietetic services. This includes resourcing dietitians with a digital platform, incorporating an image-based dietary assessment and tailored feedback system. This research could enhance the capability and capacity of dietitians by improving the effectiveness of dietetics weight management services, resourcing dietitians with an innovative, time-saving digital platform that will provide a comprehensive state-of-the-art technology in dietary assessment and feedback. This study will provide the first test of the technology alongside dietetic counseling conducted by videoconferencing in people living with obesity in Australia.

Clinical practice guidelines for the management of obesity have focused on reducing energy intake [62]. While this approach is effective, the “thinking beyond calories” concept indicates that various dietary components and patterns may negatively affect energy balance leading to obesity [63] and recidivism post intervention. This novel concept is supported by a crossover trial where inpatients were fed ad libitum either an ultraprocessed or an unprocessed diet [41]. Energy intake was greater during the ultraprocessed diet, suggesting improving diet quality and reducing consumption of ultraprocessed foods may play an important role in energy balance. Dietitian-led interventions lead to statistically significant changes, but the effect sizes do not always show clinically significant improvements in health outcomes [11]. This may be due to the inconsistent use of behavior change techniques in dietitian-led interventions. Their focus on education alone may be insufficient to facilitate behavior change [64]. For dietary interventions, the inclusion of behavior change techniques is more effective for improving health outcomes [65]. However, many studies to date have not specified the behavioral theory used nor have they measured changes in psychological variables. This study will address this gap through the comprehensive assessment of both dietary and psychological parameters, to comprehensively test the effectiveness of behavior change strategies for weight management.

### Strengths and Limitations

A strength of the study is the intervention design comparing a dietitian-led intervention with minimal intervention control. The intervention is underpinned by the COM-B model, delivered by dietitians trained in behavior change techniques [49]. In addition, the study will use the electoral roll, a compulsory enrollment system for Australians aged more than 18 years, for the recruitment of participants to provide representation across socioeconomic groups. The results will be potentially useful in clinical decision-making to inform weight management advice. A limitation is the self-selecting sample, meaning that the findings may not be generalizable to the wider population or priority groups. Therefore, the findings of this study may not be generalizable outside the Australian context, and application of the findings across different cultures may be limited. Furthermore, there may be challenges in recruiting volunteers to meet recruitment targets and ensuring representation from a diverse background for socioeconomic status and ethnicity.

### Conclusions

This research aims to improve outcomes for people living with obesity and addresses a gap in telehealth dietetics services for the delivery of weight management care. This RCT will compare a 12-month, digitally tailored, feedback dietary intervention with standard care in people living with obesity, where the feedback uses new cutting-edge technologies to automate an otherwise lengthy process. Consistent with a patient-centered model of care, the chat2 intervention incorporates video counseling by trained dietitians to support dietary behavior change and relapse prevention. This model of care will build capacity for dietitians to deliver effective evidence-based weight management advice using new technologies, potentially creating a paradigm change in the management of obesity, a common condition that is associated with a substantial disease burden.

## Abbreviations

ADG: Australian Dietary Guidelines
chat2: Connecting Health and Technology 2
COM-B: capability, opportunity, motivation, and behavior
CONSORT: Consolidated Standards of Reporting Trials
COREQ: Criteria for Reporting Qualitative Research
DQS: diet quality score
DXA: dual-energy absorptiometry
EMHS: East Metropolitan Health Service
mFR: mobile Food Record
PNQ: Personalized Nutrition Questionnaire
RCT: randomized controlled trial

## Data Availability

The datasets generated and analyzed during this study will be available from the corresponding author on reasonable request.
